# Perceptions of nephrology among medical students and internal medicine residents: a national survey among institutions with nephrology exposure

**DOI:** 10.1186/s12882-019-1289-y

**Published:** 2019-04-29

**Authors:** Devika Nair, Kurtis A. Pivert, Adrian Baudy, Charuhas V. Thakar

**Affiliations:** 1 Division of Nephrology and Hypertension, Vanderbilt University Medical Center, 1161 21st Avenue South, Medical Center North S-3223, Nashville, TN 37232-2372 USA; 2 American Society of Nephrology, Washington, DC, USA; 3 Tulane University, New Orleans, LA USA; 4 University of Cincinnati, Cincinnati, OH USA

## Abstract

**Background:**

Fewer trainees are choosing to pursue nephrology. Only 60.1% of positions filled in the 2018 fellowship Match, which is concerning given the rising prevalence of end-stage kidney disease. Identifying factors influential in career choices is critical to inform focused approaches to recruit qualified applicants.

**Methods:**

To understand perceptions of nephrology and assess factors influential in specialty choice among early career trainees, an anonymous survey was distributed to upper-level medical students and internal medicine residents at programs identified through the American Association of Medical Colleges (AAMC) and American Medical Association’s Fellowship and Residency Electronic Interactive Database (FREIDA).

**Results:**

Of 4199 recipients, 644 (15.3%) participants responded, including 315 upper-level medical students, 308 residents, and three chief residents from 30 institutions. An interest in the subject was the most critical factor in selecting a specialty (92%). Other key factors included a suitable work-life balance (73%), access to mentors (70%), and subject exposure (66%). Lack of interest was the most frequently-cited reason to forgo a nephrology fellowship (79%), followed by concerns regarding remuneration (43%), work-life balance (39%), and subject exposure (32%). In free-text responses, several participants described frustration with managing patients on hemodialysis and desired combined training with specialties such as critical care. Respondents who had considered nephrology at any point cited an interest in physiology or interface with a mentor as key driving factors.

**Conclusions:**

A lack of interest in and exposure to the subject, perceptions of poor earning potential and patient nonadherence, and concerns regarding work-life balance were influential in participants’ decisions to forgo nephrology training. Incorporating novel educational tools and broadening the scope of the nephrology elective, highlighting ongoing areas of clinical and research innovation, expanding opportunities for interdisciplinary collaboration and procedural skills, and cultivating strategies to reduce burnout may be useful areas on which to focus future recruitment efforts.

**Electronic supplementary material:**

The online version of this article (10.1186/s12882-019-1289-y) contains supplementary material, which is available to authorized users.

## Background

Currently, over 40 million individuals in the United States suffer from kidney diseases [1–3]. Given the increasing burden of disease, aging population, and declining death rate among end-stage kidney disease (ESKD) patients, the decline in nephrology fellowship applicants since 2009 poses a threat to our ability to adequately care for this population [4–6]. According to an American Society of Nephrology (ASN) analysis, only 60.1% of available nephrology fellowship positions filled in the 2018 National Resident Matching Program (NRMP) Match, and 128 fellows received their positions during the post-Match period [4]. These developments have raised concerns about candidate quality and motivation, the recruitment process, and the future of the specialty overall.

Prior studies which sought to identify reasons for the declining interest in nephrology have focused on trainees who had already chosen a subspecialty fellowship. These studies have pointed to fellows’ concerns regarding the medical complexity of nephrology as well as perceptions of limited long-term income potential as reasons for a lack of interest in choosing the field [7, 8]. To our knowledge, early career trainees have never been directly queried regarding their perceptions of nephrology and their considerations when choosing subspecialty training. As such, nephrology fellowship programs and national organizations are less equipped to develop and deliver targeted recruitment initiatives to attract strong applicants to the field. In our multi-institutional survey of upper-level medical students and internal medicine residents, we aimed to a) uncover what factors were most influential in choosing a subspecialty and b) identify which of those factors, if any, deterred applicants from choosing nephrology. We also sought to gain further insight into applicants’ answers by allowing for free-text responses. Although our analyses were exploratory in nature, we hypothesized that mentorship and concerns about income potential would emerge as two of the most influential factors in choosing or forgoing a nephrology fellowship. In performing this work, we hoped to provide insight into applicants’ perceptions of nephrology during a formative time in their career development and provide an outline of areas on which to strengthen existing recruitment efforts and develop new initiatives.

## Methods

Our survey tool was informed by prior existing analyses of fellow perceptions of nephrology and other subspecialty fields [7–9]. Specific question items were developed by the primary author and informed by two focus groups of six medical students and 15 internal medicine residents, respectively. A purposive sampling technique was employed to ensure the ethnic, gender, and age-related diversity of focus group participants. Survey items were pilot-tested twice within each focus group before questionnaire finalization (Additional file 1: Survey Tool). Apart from institution and trainee year, no identifying demographic characteristics, including visa status, were included in the survey to ensure anonymity and encourage survey participation. The research and survey tool were deemed exempt by Tulane University’s Institutional Review Board, and the survey was disseminated in May and June of 2016 using a secure, online platform (SurveyMonkey). The survey audience was identified using publicly available data sources per the following inclusion criteria: 1) allopathic medical schools listed in the American Association of Medical College (AAMC) database; and 2) internal medicine residencies listed in the American Medical Association’s Fellowship and Residency Electronic Interactive Database (FREIDA) located at institutions with an associated nephrology fellowship.

Respondents were asked to weight factors on a four-point Likert scale according to their degree of influence in the decision to pursue a specialty and which of those factors, if any, impacted the decision to forgo nephrology. Participants were given the opportunity to provide free-text responses to elaborate on whether they had ever considered a career in nephrology and share any other thoughts regarding the field. Participation was completely voluntary, respondents could skip any question of their choosing, and no incentives for participation were offered.

Additional subgroup analyses stratified by trainee level were conducted using a chi-square test for goodness of fit (alpha < 0.01) using R (https://www.r-project.org/). Open-ended responses were reviewed individually for sentiment and analyzed using the tidytext R package (https://cran.r-project.org/web/packages/tidytext/index.html) to identify frequent terms among groups.

## Results

**Fig. 1:**
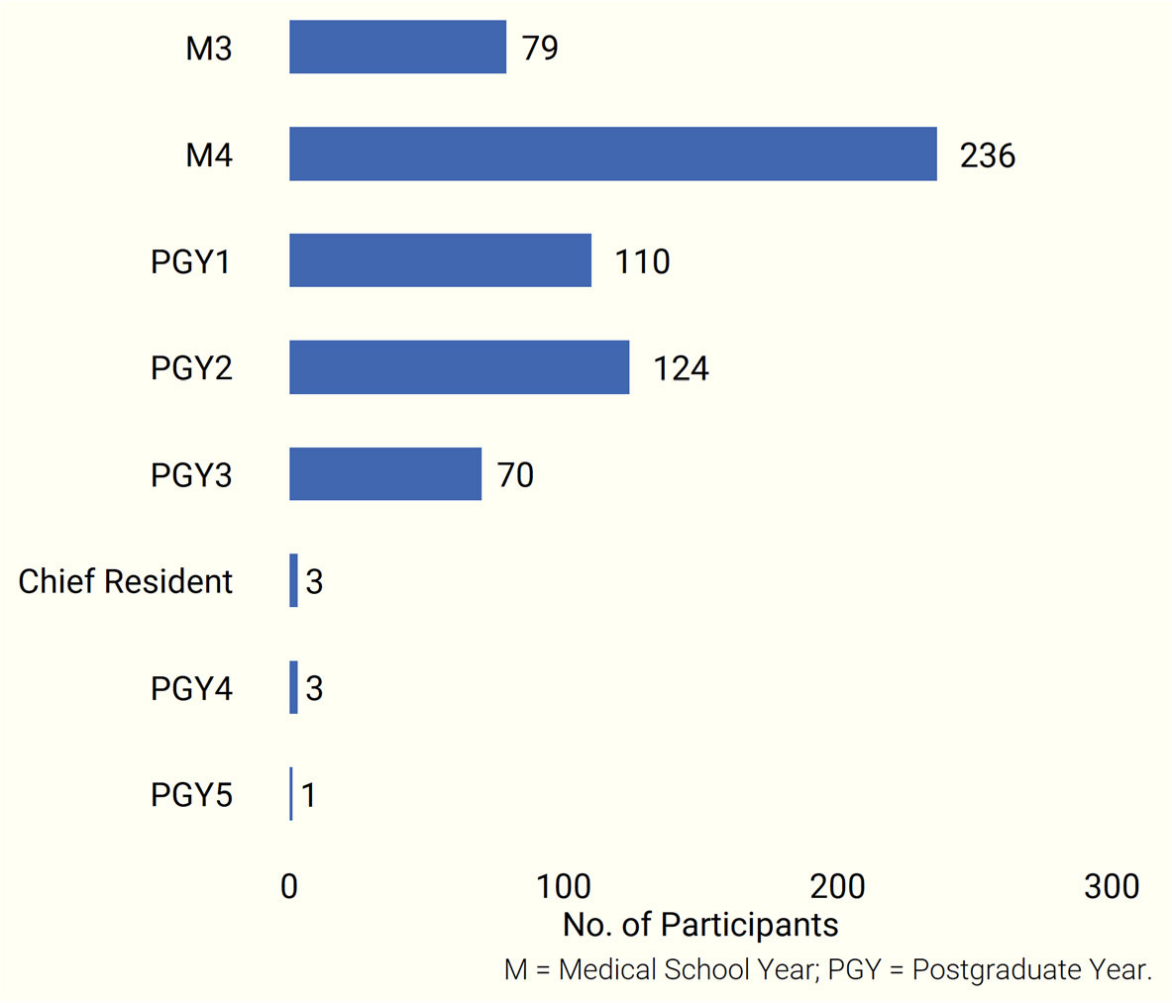
Educational status of participants

7Out of 4199 survey recipients, a total of 644 (15.3%) trainees responded (Additional file 2: Survey Responses Part 1; Additional file 3: Survey Responses Part 2). Participants were composed of 315 upper-level medical students, 308 residents, and three chief residents from 30 medical schools and internal medicine residencies. Lower-level medical students and those who did not identify their level of training (18 participants) were excluded from the analysis (Fig. 1).

### Choosing to pursue a subspecialty fellowship

**Fig. 2:**
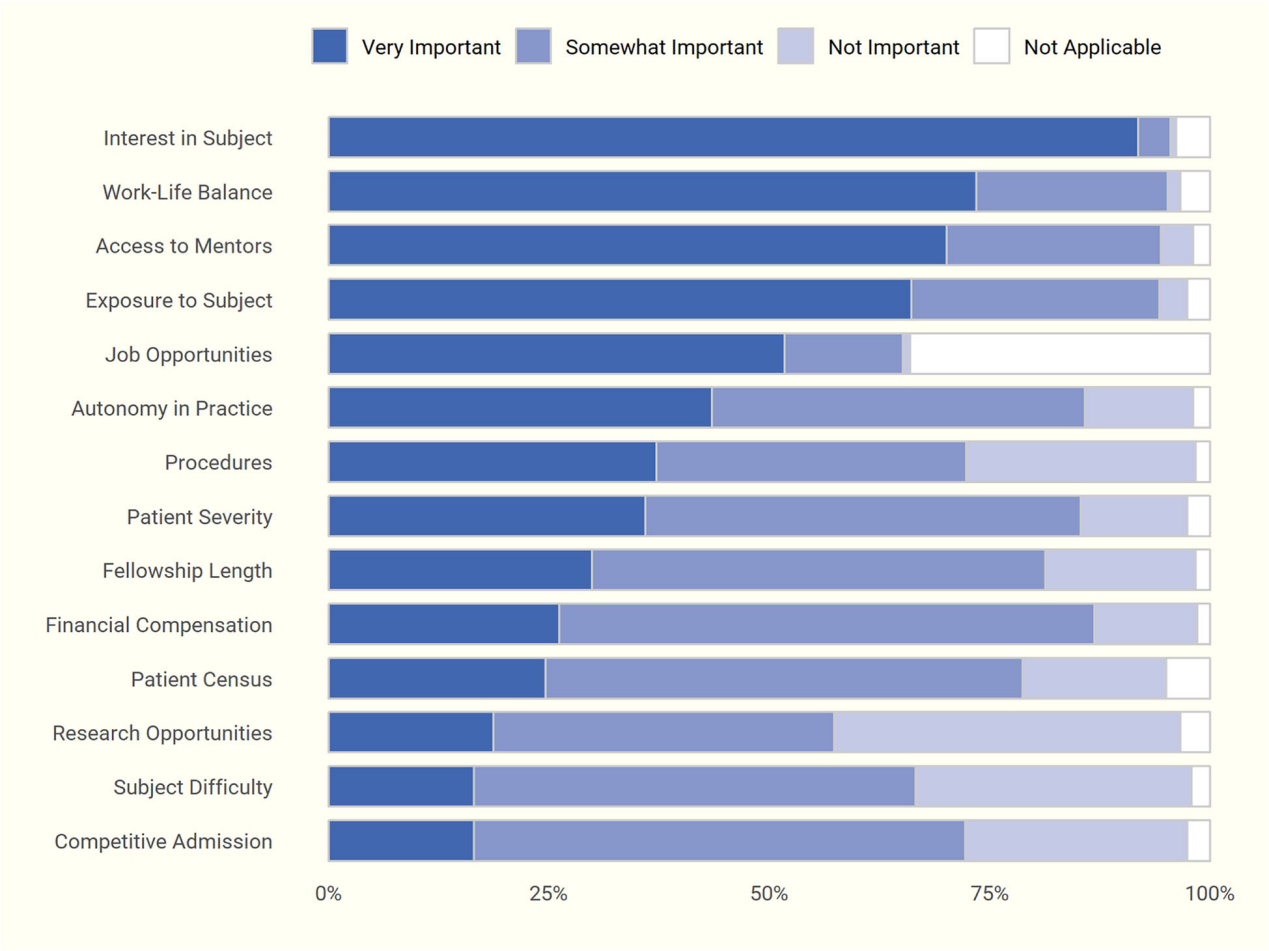
Factors influential in choosing a specialty

**Fig. 3:**
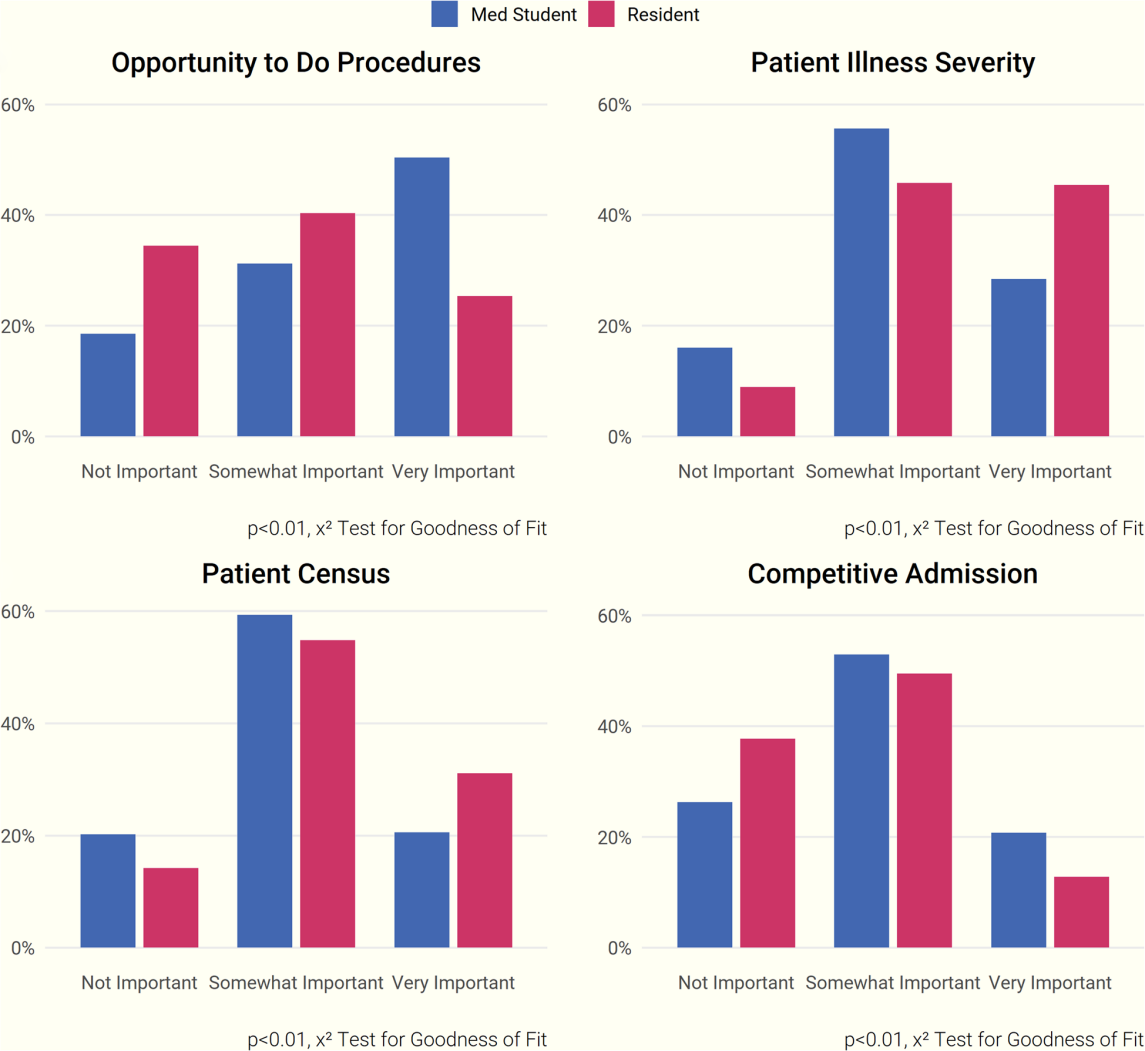
Factors influential in specialty choice, stratified by trainee level.* Blue Bars = *Medical Students; *Red Bars* *=* Residents

Respondents indicated that a personal interest in a specialty was the most influential reason for choosing to pursue it—92% of participants ranked this factor as “Very Important.” Other influential factors included a suitable post-fellowship work-life balance (73% of respondents), access to high-quality mentors (70%), and adequate exposure to the specialty prior to applying (66%). Post-fellowship research opportunities were considered the least important factor (only 19% rated it as “Very Important”) (Fig. 2). Competitiveness of admission was not influential in choosing a fellowship in our overall participant sample, but when we conducted additional analyses stratified by trainee level, a higher percentage of medical students rated competitiveness of admission as “Very Important.” Responses stratified by level of training also differed significantly on a number of other factors (*p* ≤ 0.01; Fig. 3). A higher percentage of medical students rated the opportunity for procedures as “Very Important” when choosing a specialty, while patient illness severity was more influential to residents.

### Choosing to forgo or pursue a nephrology fellowship

**Fig. 4:**
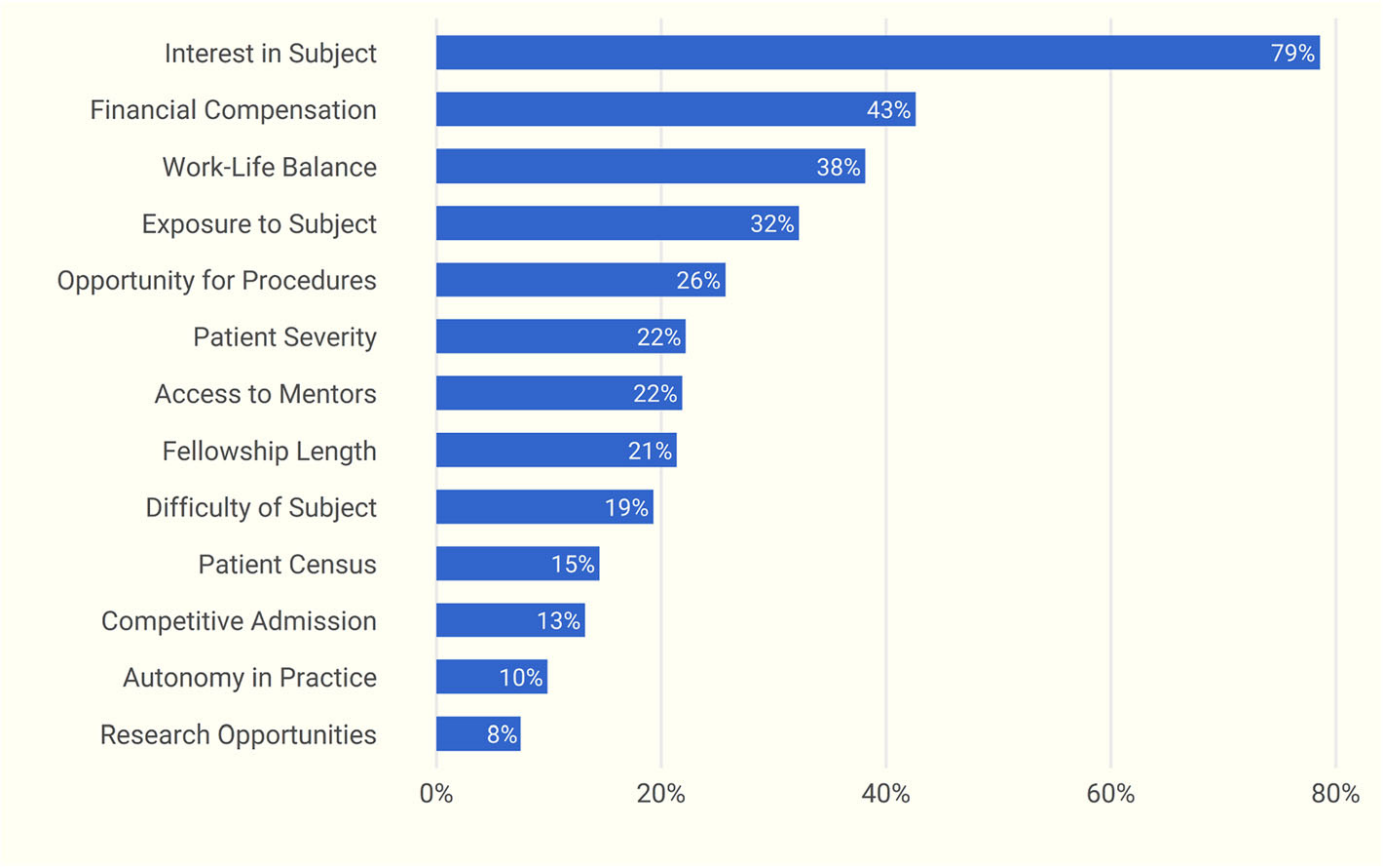
Factors influential in forgoing a nephrology fellowship

**Fig. 5:**
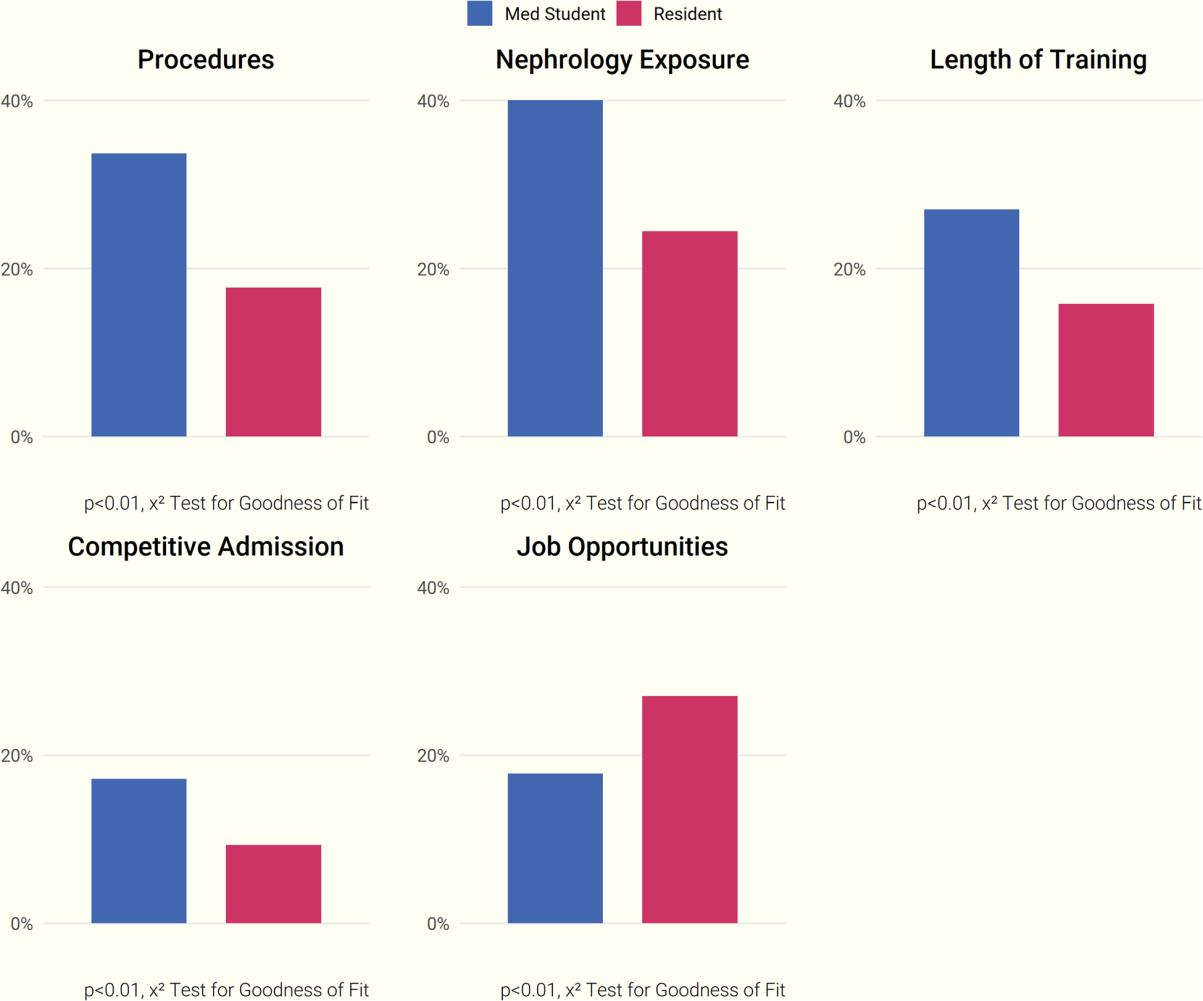
Factors influential in forgoing nephrology, stratified by trainee level. *Blue Bars*= Medical Students; *Red Bars*= Residents

Seventy-nine percent of participants cited a lack of interest as the most substantial reason to forgo nephrology fellowship training. This was followed by concerns regarding adequate financial compensation after fellowship (43%), perceptions of an unsatisfactory work-life balance (38%), and inadequate exposure to the specialty during earlier career development (32%). (Fig. 4). Medical student and resident responses differed significantly in multiple areas (*p* ≤ 0.01, Fig. 5). A greater proportion of residents considered the availability of post-fellowship job opportunities as a key driving factor in choosing or forgoing nephrology, while more medical students placed an emphasis on fellowship competitiveness, length of training, subject exposure, and the opportunity for procedures.

### Resident subgroup analyses

Concerns regarding poor financial compensation are frequently cited among prospective applicants as reasons to forgo nephrology training. A subgroup analysis of resident respondents who placed the strongest emphasis on financial compensation was conducted to identify other factors influential to these participants. Responses among those residents who indicated that compensation was ‘Very Important’ in choosing a subspecialty (91 residents) were further stratified by whether they would or would not consider nephrology. Among those who placed strong emphasis on financial compensation but stated that they would consider nephrology (26 residents), 46% indicated that the ability to perform procedures was ‘Very Important’ when considering a subspecialty. Other factors labeled ‘Very Important’ by this group included patient illness severity (53.8%), patient census (62%), access to mentors (69%), and post-fellowship autonomy (50%). Among those who strongly emphasized financial compensation but indicated that they would forgo nephrology training (65 residents), 35% placed the strongest emphasis on patient illness severity, 35% on patient census, 59% on access to quality mentors, and 43% on post-fellowship autonomy.

### Free-text responses and themes

**Fig. 6:**
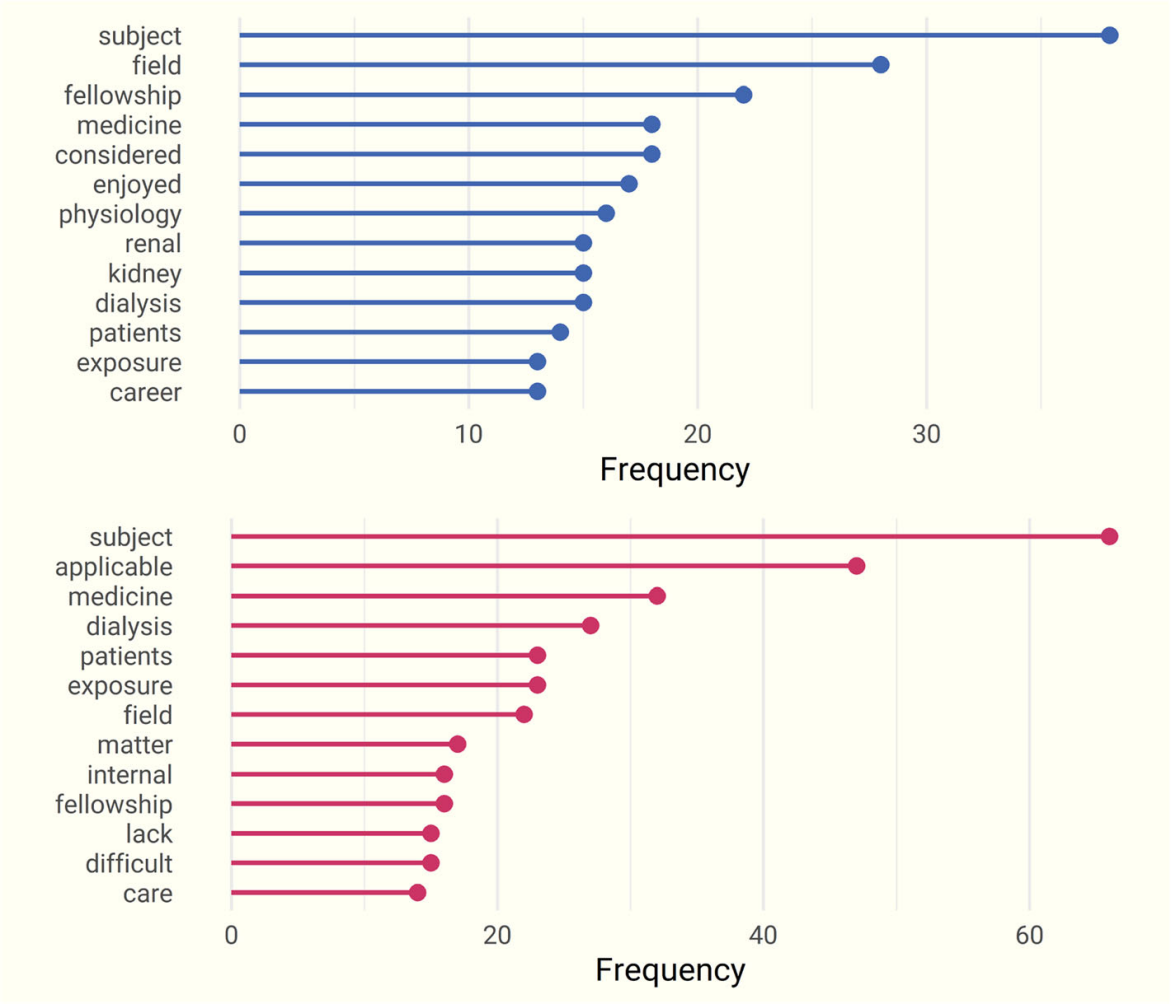
Most frequent terms identified in the free-text analysis for respondents who would *(upper)* and who would not *(lower)* consider a career in nephrology

Open-ended responses were reviewed using a manual sentiment analysis and subsequently analyzed using a bag-of-words model (Fig. 6). “Subject,” “enjoyed,” and “physiology” were consistently noted among respondents who stated that they would consider careers in nephrology. Those participants who wished to pursue other specialties also used “subject” in their responses, but this was accompanied by the words “dialysis,” “exposure,” “lack,” and “difficult.” Fifty-five participants (8%) found the prospect of managing chronic dialysis patients daunting and cited perceived patient nonadherence as a specific concern. Thirteen participants indicated that a combined nephrology–critical care fellowship would have considerable appeal. Other informative free-text responses are highlighted below.

Reasons to forgo a nephrology fellowship:

*“Nephrology fellows seem busy and unhappy.”*

*“All your patients are on dialysis and that’s the endpoint. You are not saving anyone’s life… you are just prolonging it.”*

*“Finding jobs that waiver visa issues is difficult. You may end up choosing a hospitalist position despite feeling over-qualified.”*

Reasons to pursue a nephrology fellowship:

*“It’s an interesting field, and you [perform] kidney biopsies!”*

*“Great mentors. Interesting physiology.”*

*“My father had a kidney transplant, and I am currently working on a research project related to transplantation.”*

Interestingly, 154 participants (75 upper medical students, 76 residents, and all three chief residents) revealed that they had considered a career in nephrology at some point. While five of these participants specifically mentioned pursuing critical care, the ultimate career paths of the other participants were not revealed. Twenty-three of these respondents specified an interest in renal physiology as their reason for considering nephrology, and 21 mentioned having had interface with excellent mentors early in their training.

## Discussion

**Fig. 7:**
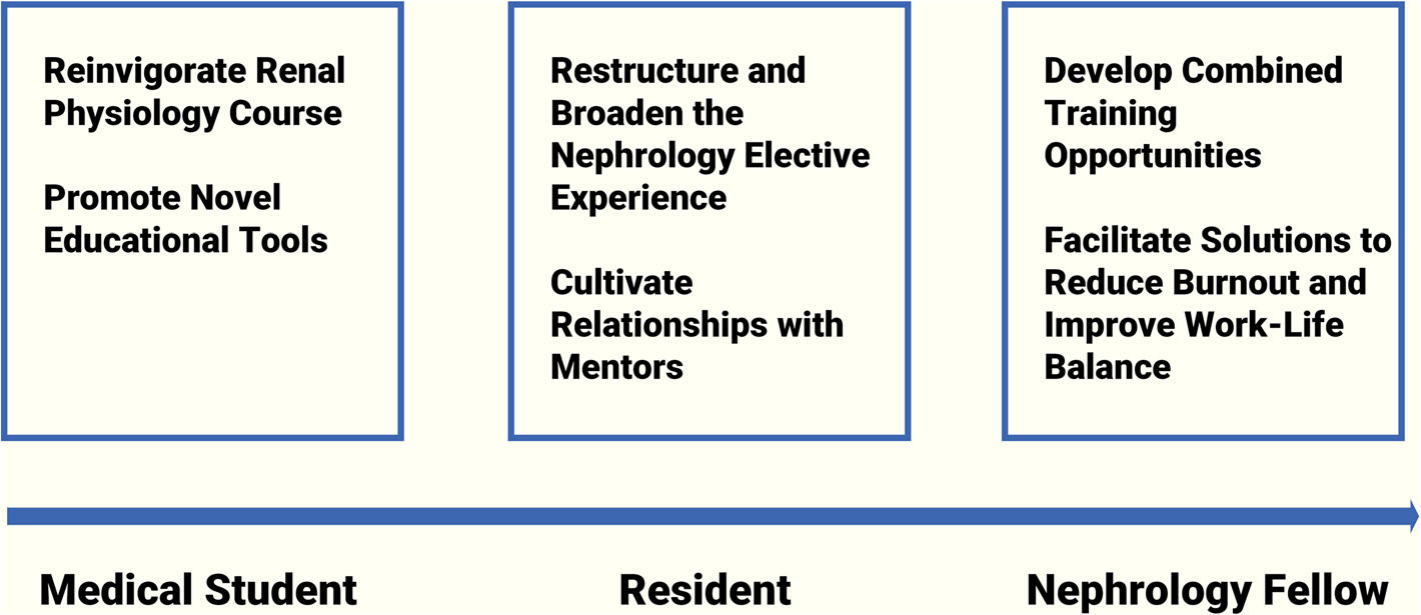
Strategies to improve recruitment along the nephrology trainee continuum

This survey builds upon prior work in this area and adds new information to what is known regarding trainee perceptions of nephrology overall and has several strengths. To our knowledge, it is the first analysis of its kind that specifically queries trainees at an earlier stage in their career development to identify perceptions of nephrology. The external validity of our survey is strengthened by the wide geographical distribution and level of National Institutes of Health funding of our 30 participating institutions. Our survey also uniquely allowed for open-ended responses, which provided an additional layer of insight into specific aspects of nephrology that deterred or attracted prospective applicants. Informed by our results, we summarize strategies to bolster recruitment in Fig. 7 and pinpoint ongoing recruitment efforts and areas for future focus in Table 1. Our rationale for these recommendations is described below.

**Table 1:** Ongoing efforts and suggestions for future areas of focus

Area of Concern	Ongoing Efforts	Potential Future Areas of Focus
Interest in subject matter	Kidney STARS and Kidney TREKS	Team-based learning and interactive renal physiology modules
	KidneyCon^a^	Educational videos and mobile applications for renal physiology
	ASN Innovations in Education contest	Podcasts about nephrology careers
	NephJC, NephMadness^b^	
Nephrology elective experience	Outpatient dialysis elective	Elective in transplantation
		Exposure to critical care, interventional nephrology, and palliative care
Mentorship	ASN Mentor-Mentee online curriculum	Formal mentorship curriculum for faculty
	Career Advancement ASN Communities forum	
	Kidney STARS and Kidney TREKS	
Combined training opportunities	Programs which offer sequential critical care and nephrology training^c^	Additional combined training with critical care, interventional radiology, or geriatrics/palliative care
Job opportunities and financial compensation	Improved J1 waiver job placements	Procedural skills training
	Hospital-based or dialysis organization-based employments	Advanced career pathways and collaboration across disciplines
Work-life balance and burnout	Restructured fellow call schedules	Reduction of the electronic health record burden
	Organizational platforms for open discussion and advocacy	Development of improved comprehensive care models
		Continued organizational initiatives to foster culture change

Abbreviations: American Society of Nephrology (ASN), Students and Residents (STARS), Tutored Research and Education for Kidney Scholars (TREKS)

^a^Annual educational conference for trainees held in Little Rock, AR (http://kidneycon.org)

^b^Annual online educational competition (https://www.tourneytopia.com/ajkd/nephmadness)

^c^University of Texas at Southwestern, Mayo Clinic, University of Kentucky, Henry Ford Health System, et al.

### Interest in the subject matter

Our results support that an interest in nephrology should be fostered early in a trainee’s career, as medical students listed subject exposure as highly influential. One way to foster early interest is to ensure the introductory renal physiology course offered to students is taught by dynamic and engaged instructors. Interactive web-based games and team-based learning modules, the latter of which have demonstrated significant knowledge gains among medical students, can be utilized more frequently [10, 11]. The ASN Kidney TREKS (Tutored Research and Education for Kidney Scholars) and Kidney STARS (Students and Residents) programs, which provide opportunities for intensive learning experiences and help identify ongoing clinical and research innovations to trainees, should continue to be promoted to trainees who demonstrate an early interest in the specialty [12]. In a preliminary return on investment analysis, 48% of participants of these programs were found to subsequently enter a pipeline specialty leading to nephrology or were conducting kidney-disease related research [13]. Educational tools that leverage social media’s broad reach, such as the NephJC Twitter-based journal club, have been gaining increased attention for facilitating open and informative discourse between nephrology thought leaders and trainees alike [14]. These and other educational tools such as mobile applications and podcasts should continue to be developed and promoted, but additional studies are needed to comprehensively assess different educational methods and their effectiveness across trainee stages.

### Nephrology elective experience

The resident experience during a nephrology rotation is a valuable opportunity to engage trainees, and nephrologists’ capability to manage patients across the outpatient, inpatient, post-transplantation, and critical care spectrum may not be well-known to the early learner. In one survey of internal medicine residents at an academic tertiary care center, residents specifically expressed a desire to have more interface with nephrologists in care settings apart from the inpatient consult service [15]. In the free text responses of our survey, the fourth most frequent response term (in 7% of responses) was ‘dialysis.’ Participants cited the obligation to manage chronic hemodialysis patients as a dissuading factor in pursuing nephrology fellowship training, a response likely driven by prior experiences on an inpatient consult service. Nephrologists are responsible for a large portion of the primary care needs of their patients. An outpatient hemodialysis rotation that includes some home hemodialysis exposure could overcome these negative perceptions as well as attract applicants who seek longitudinal patient relationships in a primary care setting [16]. As a number of medical schools and residencies have successfully incorporated outpatient dialysis exposure into their nephrology electives, adding transplantation and critical care exposure may be the next step to widen the appeal and strengthen the utility of the nephrology elective experience [17].

### Mentorship

Out of 154 survey participants who expressed an interest in pursuing nephrology, 12 (8%) cited mentorship as a highly influential factor in making this choice. It has been well established that mentorship is critical to a trainee’s success, and suitable mentors provide education, guidance, constructive criticism, and honest feedback to mentees [18]. Trainees who demonstrate an early interest in nephrology should be directed to potential faculty mentors with similar clinical and research interests. ASN’s recently-launched online mentoring curriculum contains helpful, user-friendly resources to help mentors and mentees set clear goals and expectations, troubleshoot roadblocks, and work together to maximize collaboration and productivity [19]. Formal mentorship training may also be beneficial for faculty members to strengthen their skills to ensure both mentor and mentee success [20]. Deliberate institutional planning may be needed to develop mentor relationships customized to trainees according to their educational stage, and partnerships between internal medicine and nephrology training program directors may help facilitate these initiatives.

### Combined fellowship training

Numerous participants in our survey expressed a strong interest in combined fellowship training with nephrology, citing critical care as a key area of interest. According to recent Electronic Residency Application Service (ERAS) data, more nephrology applicants concurrently apply for a pulmonary-critical care fellowship than any other subspecialty [21]. Continuing to develop programs which offer combined fellowship training in nephrology and an added subspecialty may help increase the breadth of the trainees we attract as well as broaden the clinical and procedural skills of nephrology fellows [22, 23]. Such developments would require long-term planning, awareness of fellows’ visa requirements, collaboration across disciplines and within institutions, and dialogue on a national level to identify training partnerships with both educational value and strong employment prospects.

### Job opportunities and financial compensation

Resident respondents of our survey were particularly concerned with a perceived lack of post-fellowship job opportunities. Thankfully, ASN’s recent workforce survey points to a decline in the percentage of fellows who had trouble securing a satisfactory position—38% in 2018 as compared to 53.1% in 2016. Additionally, fewer international graduates are struggling to find post-fellowship employment, as 55.4% reported difficulty in 2018 as compared to 70% the year prior [24].

Though the American Medical Group Association (AMGA) reported that nephrology was among the subspecialties with the largest increase in financial compensation, and nephrologists have been shown to have a higher earning potential than hospitalists, concerns about remuneration were widespread among our survey participants [25, 26]. One way to increase opportunities for compensation is to foster to the development of new skills. Point-of-care ultrasonography has been gaining increased attention for its applications across the spectrum of nephrology care [27]. While these procedures are operator-dependent and need to be tested for inter-observer reliability and accuracy, fellowship programs have begun to incorporate point-of-care ultrasonography into their didactic curriculum [28]. Developing opportunities for combined training across disciplines such as critical care and interventional radiology may also offer additional venues for skill development and opportunities for compensation. Ultimately, the competitiveness of a specialty drives compensation, and ensuring that nephrology remains both competitive and attractive is critical to facilitating adequate remuneration for practice.

### Work-life balance

Survey participants cited work-life balance as a key determinant in choosing a specialty. Nephrology fellows are often among the busiest in the hospital, and to many applicants, nephrologists are perceived to lead demanding and unsatisfying lifestyles. Burnout has been increasingly recognized and described within the nephrology community [29]. While incorporating nurse practitioners and physician assistants to provide comprehensive care have been effective in reducing workload, organizational interventions to increase physician autonomy and restore a sense of purpose must continue to be created. ASN is currently in the process of developing sustainable ways to address burnout at a training program, organizational, and national level, including working with the Centers for Medicare and Medicaid Services to reduce the electronic health record burden and develop comprehensive models that deliver high-quality, patient-centered kidney care [30].

As with all survey-based research, this study had a number of limitations, including self-selection and non-response biases. Only residencies with affiliated nephrology fellowships were selected for inclusion, which potentially excluded many trainees interested in nephrology and limits the generalizability of our results. Only those factors mentioned in the survey could be chosen as reasons for pursuing or not pursuing nephrology, though opportunities for open-ended responses were provided. We do not know the ultimate career decisions of participants who initially favored nephrology but went on to choose other specialties, though an interest in critical care was commonly cited. To maintain the anonymity of participants, certain demographic variables that may otherwise have been informative were not collected, such as gender, and institution-specific responses were not analyzed. Participants were also not asked to specify their visa status, a factor known to influence post-fellowship job opportunities and locations of practice.

## Conclusions

Based on our analyses, a personal interest in a specialty is the prime motivator for medical students and internal residents in choosing a career path, followed by work-life balance and mentorship access. A lack of interest was the most-cited factor for participants to forgo nephrology fellowship training, followed by perceptions of inadequate financial compensation and poor work-life balance. Potential ways to increase interest in nephrology include adopting innovative teaching methods focused on practical applications of renal physiology, exposing trainees to research and clinical innovation, restructuring the nephrology resident elective experience, facilitating relationships with suitable mentors, increasing opportunities for combined fellowship training, finding ways to broaden nephrologists' clinical and procedural skills, and developing methods to foster a suitable work-life balance and reduce burnout. Sustaining a passionate and dynamic nephrology workforce is contingent upon clinicians, educators, and researchers to highlight ongoing innovations, develop new initiatives, and continue to be ambassadors for the field overall.

## Additional files

Additional file 1: Survey Tool. Survey questions.

Additional file 2: Fellowship Interest Questionnaire Part 1. First data file from survey responses.

Additional file 3: Fellowship Interest Questionnaire Part 2. Second data file from survey responses.
