# Supplementary material for: Perceptions of nephrology among medical students and internal medicine residents: a national survey among institutions with nephrology exposure
Source: BMC Nephrol. 2019 Apr 29;20:146. doi: 10.1186/s12882-019-1289-y (PMC6489240; doi:10.1186/s12882-019-1289-y)
Supplement: Supplementary file 1 — Survey Tool. Survey questions. [file 12882_2019_1289_MOESM1_ESM.docx]

**Appendix: Survey Tool**

1. What is your institution?

2. What is your current level of training (3rd-year medical student, 4th-year medical student, intern, resident, etc.)?

3. Please comment on each of the following when considering a specialty fellowship:

Not Important Somewhat Important Very Important Not Applicable


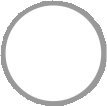

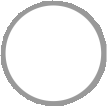

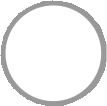

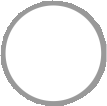


Financial compensation


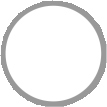

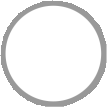

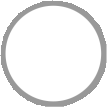

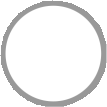
Opportunity to do procedures


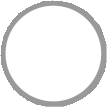

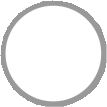

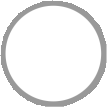

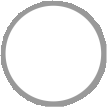


Adequate exposure to subject

Patient severity/illness
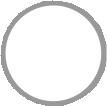

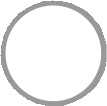

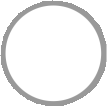

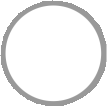


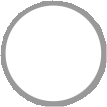

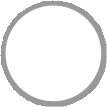

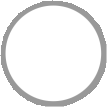

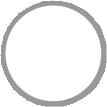


Patient census


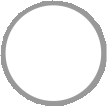

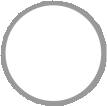

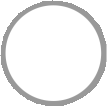

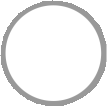
Length of fellowship training


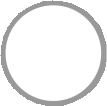

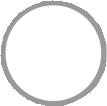

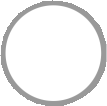

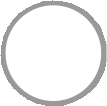


Competitiveness of admission


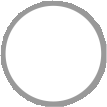

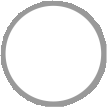

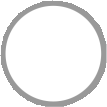

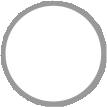
Work-life balance post- fellowship


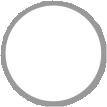

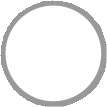

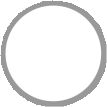

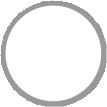


Interest in subject


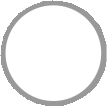

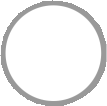

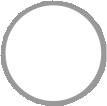

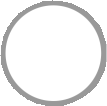
Access to high-quality mentors


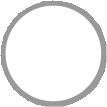

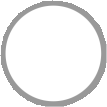

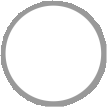

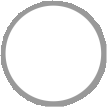


Difficulty of subject


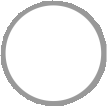

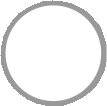

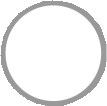

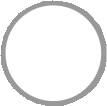
Autonomy in practice post-fellowship


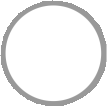

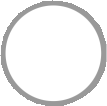

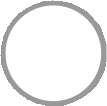

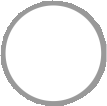


Research opportunities during and post- fellowship


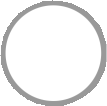

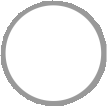

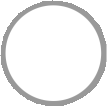

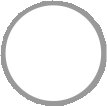
Job opportunities post- fellowship

4. Which of the following (if any) may influence your decision to not pursue nephrology? Please select all applicable answers.

Financial compensation post-fellowship

Opportunity to do procedures during and after fellowship

Adequate exposure to subject prior to and during residency

Patient severity/illness

Patient census

Length of fellowship training

Competitiveness of fellowship

Work-life balance post-fellowship

Interest in subject

Access to high-quality mentors

Difficulty of subject

Autonomy in practice post-fellowship

Research opportunities during and post-fellowship

Job opportunities post-fellowship

5. Have you ever considered a career in nephrology? Why or why not?

6. Please enter any additional thoughts on this subject here. Thank you for your participation and help!
